# Automated Versus Manual Blood Pressure Measurement: A Randomized Crossover Trial in the Emergency Department of a Tertiary Care Hospital in Karachi, Pakistan: Are Third World Countries Ready for the Change?

**DOI:** 10.3889/oamjms.2016.076

**Published:** 2016-07-27

**Authors:** Kanaan Mansoor, Saba Shahnawaz, Mariam Rasool, Huwad Chaudhry, Gul Ahuja, Sara Shahnawaz

**Affiliations:** 1 *Dr. Ziauddin University Hospital, Karachi, Pakistan*; 2 *Dialysis Unit, The Kidney Centre, Karachi, Pakistan*; 3 *Sindh Government Hospital, Karachi, Pakistan*; 4 *New Mehran Medical Centre, Karachi, Pakistan*

**Keywords:** Sphygmomanometers, Automated oscillometer, blood pressure

## Abstract

**BACKGROUND:**

Hypertension has proven to be a strong liability with 13.5% of all mortality worldwide being attributed to elevated blood pressures in 2001. An accurate blood pressure measurement lies at the crux of an appropriate diagnosis. Despite the mercury sphygmomanometer being the gold standard, the ongoing deliberation as to whether mercury sphygmomanometers should be replaced with the automated oscillometric devices stems from the risk mercury poses to the environment.

**AIM:**

This study was performed to check the validity of automated oscillometric blood pressure measurements as compared to the manual blood pressure measurements in Karachi, Pakistan.

**MATERIAL AND METHODS:**

Blood pressure was recorded in 200 individuals aged 15 and above using both, an automated oscillometric blood pressure device (Dinamap Procare 100) and a manual mercury sphygmomanometer concomitantly. Two nurses were assigned to each patient and the device, arm for taking the reading and nurses were randomly determined. SPSS version 20 was used for analysis. Mean and standard deviation of the systolic and diastolic measurements from each modality were compared to each other and P values of 0.05 or less were considered to be significant. Validation criteria of British Hypertension Society (BHS) and the US Association for the Advancement of Medical Instrumentation (AAMI) were used.

**RESULTS:**

Two hundred patients were included. The mean of the difference of systolic was 8.54 ± 9.38 while the mean of the difference of diastolic was 4.21 ± 7.88. Patients were further divided into three groups of different systolic blood pressure <= 120, > 120 to = 150 and > 150, their means were 6.27 ± 8.39 (p-value 0.175), 8.91 ± 8.96 (p-value 0.004) and 10.98 ± 10.49 (p-value 0.001) respectively. In our study 89 patients were previously diagnosed with hypertension; their difference of mean systolic was 9.43 ± 9.89 (p-value 0.000) and difference of mean diastolic was 4.26 ± 7.35 (p-value 0.000).

**CONCLUSIONS:**

Systolic readings from a previously validated device are not reliable when used in the ER and they show a higher degree of incongruency and inaccuracy when they are used outside validation settings. Also, readings from the right arm tend to be more precise.

## Introduction

One of the most common medical tests done on thousands of patients every day is a blood pressure measurement. An accurate measurement is vital in providing appropriate treatment. It aids in the diagnosis of various conditions; ranging from dehydration in diarrhoea patients with low readings to vascular disease patients with elevated readings. A BP reading can be the defining point of treatment in patients with chest pain or altered mental status.

Hypertension is one of the leading causes of developing atherosclerosis, cerebrovascular disease, stroke, ischemic cardiac disease, congestive heart failure and myocardial infarction [1]. Elevated blood pressure is also associated with the development of renal failure and dementia [2, 3]. The prevalence of Hypertension in Pakistan was 10% in 1997 [4] while in Canada it is estimated to be 20% prevalent (that is every 1 in 5 persons has HTN) [5]. HTN related diseases are on the rise and are a global burden. According to a report published in 2008, 54% of strokes, 47% of ischemic heart diseases and 13.5% of all mortality worldwide was attributable to elevated blood pressures in 2001 [6].

Physicians are thus advised to routinely check for BP elevation in all of their patients. Overestimation of BP can expose the patient to the potential adverse effects of drug treatments as well as unnecessary medical costs and dietary restrictions. While with an underestimated reading, the patient is at a risk of HTN related diseases, which can significantly reduce life expectancy. Therefore an accurate reading is essential [7].

There are three non-invasive modalities commonly used to check blood pressure throughout the world, namely the manual mercury sphygmomanometer, aneroid meter and the automated oscillometric device. The manual mercury sphygmomanometer is considered to be the gold standard [8] that is, if used by a trained nurse or doctor. Recently, however, there is an ongoing debate about whether mercury sphygmomanometers should be replaced with the automated oscillometric devices because of health concerns. Mercury is a toxic substance and is considered an environmental hazard. It has been banned in various European countries such as Sweden and The Netherlands as well as in numerous hospitals in the United States [8, 9].

A myriad of factors can affect manual blood pressure measurements such as the site of placement of the cuff, the size of the cuff, type of stethoscope, following the proper protocol, patient’s age group, pregnancy, exercise, arrhythmias and the white coat response [10]. Readings can also vary depending on whether the nurse or the doctor is conversing while taking the measurement and whether there is background noise or silence [7, 11]. All these factors contribute towards possibly inaccurate BP readings, with a potential for misdiagnosis.

Apart from the above-mentioned causes that are mostly associated with the manual mercury sphygmomanometer, there are causes that might influence the readings of both AO BP devices and the manual BP like respiration, emotions, tobacco, alcohol, temperature, bladder distension, pain and exercise. Most of these are controllable, while some are non-modifiable like age, race and diurnal variation [12-15]. Automated Oscillometric devices are seen to be less influenced by most of these factors and recent studies indicate that they virtually eliminate the white coat response [16].

Multiple studies suggest that the AO devices should replace the conventional manual mercury sphygmomanometer, as the latter is destined to become obsolete [17, 18]. However, with limited resources and the high costs involved in attaining the latest medical equipment, a significant question arises: Are third world countries ready for the change?

This study was conducted at a tertiary care hospital in Karachi, Pakistan, using the resources at hand, with the available AO BP instruments and the mercury sphygmomanometer. The study was performed to check the validity of AOBP measurements as compared to the manual BP measurements in Karachi, Pakistan.

## Subjects and Methods

The study was conducted at a tertiary care hospital in Karachi, Pakistan. This hospital is equipped with AOBP monitors, Dinamap Procare 100 and manual mercury sphygmomanometers with a Littman classic II stethoscope. This was a double-blind randomised clinical crossover trial. Every patient was assigned two staff nurses who used an automated oscillometric blood pressure device and a manual mercury sphygmomanometer on the patient concurrently. The device, arm for taking the reading and nurses were randomly determined. Both the nurses remained blind of the readings they recorded.

All patients were in a supine position when their BP was checked. Patients aged 15 and above were included, whereas patients with complaints of chest pain, altered mental status, GCS < 12/15 and patients who had smoked within the last 30 minutes were all excluded.

Randomization was done by giving the staff nurses printed forms that they filled and then sealed in the same envelope for a given patient. The crossover was achieved by taking manual than auto or auto and then manual measurements. The form consisted of fields that required the name of the device, arm from which the reading was taken, presenting complaint, history of HTN, DM, IHD, renal diseases, neurological disorders, and chronic respiratory tract diseases.

The analysis was done using SPSS version 20. Test of significance was T test and *p-values* of 0.05 or lower were considered to be significant. Bland- Altman plots were employed to graphically represent the data. This study was done from February 2015 to May 2015. The sample size was 200.

## Results

The Emergency Department is one of the most vital departments in the hospital. Decisions made in the ED shape the rest of the therapy for the patient. EDs all over the world receive a diverse array of patients from all age groups with a variety of clinical conditions. Accurate blood pressure measurements are pivotal for treatment, especially for patients with deteriorating conditions. In 2011, a study suggested that substituting automated oscillometric devices for auscultatory devices could cause grave repercussions for patients in specific circumstances; and in cases of trauma or deteriorating patient condition, manual BP should be given preference [19]. Despite this, oscillometric devices are gaining popularity and are steadily replacing auscultatory devices [8].

Even at very low levels, environmental mercury can act as a potent neurotoxin and cause serious harm. Health care facilities are one of the main sources of mercury pollution via emissions from incineration of medical waste. Mercury sphygmomanometers, collectively, are the largest reservoir of mercury in the health care setting, often containing 80-100 g/unit. The WHO considers them a major occupational hazard, as inadequate care may result in dangerous exposures to patients and health care staff [20]. WHO and other organisations around the world are working towards the removal of Mercury from hospitals and other health care settings due to the potential threat it poses [20]. In 1998, after an agreement between the American Hospitals Association and the U.S Environmental Protection Agency, Hospitals for Healthy Environment (H2E) was launched to virtually eradicate mercury from the healthcare setup in the U.S. [21]. Many countries have already switched from mercury sphygmomanometers to alternative devices [22].

Automated oscillometric devices, on the other hand, are not only considered environmentally safe but they also have a significant advantage over their manual counterparts (mercury or aneroid): they don’t require a trained professional to take a reading. This makes them perfect for ambulatory readings for patients and to monitor BP at home. Primarily for this reason, oscillometric devices are gaining fame. A bias commonly faced by physicians is white coat hypertension, and it has been proven that automated oscillometric devices substantially remove this effect leading to more accurate readings [23, 24].

Many oscillometric devices are available in the market. Most of these devices are not put through any validation and yet they are still being sold and used by the population. Thus there is a growing concern regarding many of these devices [25, 26]. The British Hypertension Society (BHS) and the US Association for the Advancement of Medical Instrumentation have devised protocols that are widely used for validating BP monitors [27]. They have compiled a list of devices that are approved for home as well as clinical use [28]. If a device fulfils the criteria set by these two organisations it can be recommended. Unfortunately, most devices are not evaluated for accuracy independently by using these two protocols [26].

Oscillometric devices can have unreliable readings when used on diabetic patients, pregnant women, elderly patients and patients with arrhythmias [27, 29]. They have to be independently validated and also need to be calibrated at regular intervals to ensure that their readings are accurate. Oscillometric devices have been reported to overstate blood pressure and at times understate blood pressure. In both of these instances, it can put patient management at risk. It has been reported that on some devices, there is an inherent flaw in the algorithms used which leads to skipping of certain values; this can influence results [30].

In our study, the mean of difference in manual systolic and automated systolic blood pressure was 8.54 with a standard deviation of ± 9.38, while the mean of difference in manual diastolic and automated diastolic was noted to be 4.21 with a standard deviation of ± 7.88. The range of difference between manual systolic and automated systolic blood pressure was calculated to be -20 to +38, while the difference between the manual diastolic and automated diastolic blood pressure was -17 to +29. The 95% CI for systolic blood pressure lies in the range of -9.84 to +26.92.

**Figure 1:**
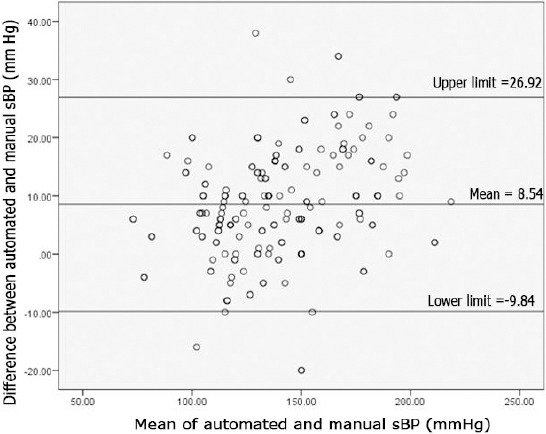
Variation in a Bland – Altman plot for difference between automated and manual systolic blood pressure when compared to the mean of diastolic blood pressure

**Figure 2:**
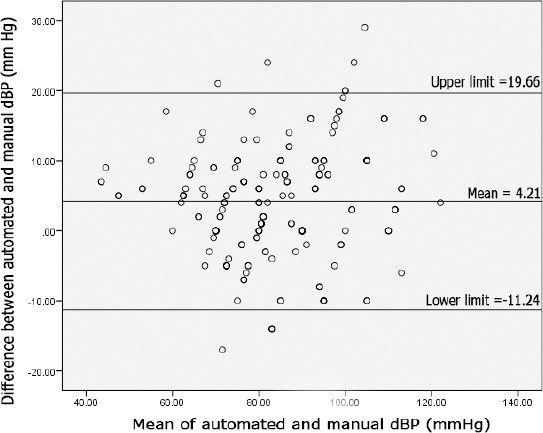
Variation in a Bland – Altman plot for difference between automated and manual diastolic blood pressure when compared to the mean of systolic blood pressure

This range of 36.76 demonstrates that automated blood pressure monitors lack precision for use at the emergency department. The 95% CI for diastolic blood pressure lies in between +19.66 to -11.24, this range of 30.9 is yet again not precise enough to yield a reliable diastolic blood pressure reading in the ED. The plots in Figure 1 and Figure 2 show that there is significant disagreement between automated and manual devices, again certifying that automated devices should not be recommended for use in the ED. Looking at the Bland-Altman plots for systolic blood pressure readings, there appears to be a positive linear trend – as the average blood pressure is rising, the difference between manual and electronic readings (subsequently called the error of electronic measurement) is also rising.

Exploring this further, we divided the patients into three groups based on their BP readings: patients who had a manual systolic reading of below 120 mmHg, those who had readings between 120 mmHg and 150 mmHg and those who had readings above 150 mmHg. The differences between manual and electronic readings for the three groups are given as follows.

A one-tailed t-test was done to see whether the observed error of electronic equipment was statistically different from the recommended AAMI criteria: mean < 5 mmHg and a standard deviation < 8 mmHg. Based on the p-values, patients who had systolic blood pressure less than 120 mmHg fell within acceptable range.

We also observed differences in measurements between the left arm and the right arm, particularly for Systolic readings. SBP measured on the right arm had less error and less standard deviation as compared to measurements on the left arm.

Analysing this further, we checked whether the error component of the electronic device was varying based on the arm of measurement and the actual (manual) blood pressure readings. One-tailed t-tests were done to check if each group was statistically different from the recommended AAMI criteria (mean of < 5 mmHg and standard deviation of < 8 mmHg).

The null hypothesis was that the observed error is not different from the AAMI criteria. Where the null hypothesis was rejected, the p-values have been marked in red, which indicate that there is a difference between the observed error and the AAMI criteria.

From this table (Table 3) it can be seen that the error difference of diastolic readings is falling within the acceptable range under all conditions. However, the error in systolic readings is only acceptable when taken on the right arm and when the measurement is below 150 mmHg.

**Table 1:** Differences between manual and electronic readings for the three groups

Systolic BP	Mean Difference Electronic and Manual	N	Std. Deviation	p-values
<= 120	6.27	73	8.39	0.175
>120 & <=150	8.91	70	8.96	0.004
>150	10.98	57	10.49	0.001
Total	8.54	200	9.38	

**Table 2:** Differences in measurements between the left arm and the right arm

Right Arm	N	Mean		Std. Deviation
Difference in Systolic	102	7.3333	0.87017	8.78831
Difference in Diastolic	102	3.6275	0.79541	8.03325

Left Arm	N	Mean		Std. Deviation

Difference in Systolic	98	9.7959	0.99409	9.84096
Difference in Diastolic	98	4.8163	0.77927	7.71442

**Table 3:** Differences in systolic and diastolic blood pressure

Blood Pressure	Arm	Difference Systolic				Difference Diastolic			
		Count	Mean	Standard Deviation	p-value	Count	Mean	Standard Deviation	p-value
<= 120	Right	42	4.02	8.24	0.71	42	3.38	6.58	0.84
	Left	31	9.32	7.73	0.02	31	6.16	5.56	0.25
>120 & <=150	Right	36	7.83	6.91	0.06	36	2.64	8.94	0.88
	Left	34	10.06	10.71	0.02	34	5.06	9.46	0.49
>150	Right	24	12.38	9.94	0.00	24	5.54	8.89	0.41
	Left	33	9.97	10.91	0.02	33	3.30	7.41	0.81

## Discussion

While some studies clearly favour oscillometric devices [17], others argue that auscultatory measurements are comparatively more accurate [18]. The inaccuracy of automated over manual monitoring has been reported with regards to the failure of automated monitoring to reliably detect orthostatic hypotension in patients at the ER in triage [31]. In our study, out of the sample of 200 patients, 89 had diagnosed hypertension. In these patients, the mean of difference of systolic blood pressure was 9.43 with a standard deviation of ± 9.89 (*p-value 0.000*), while the mean of difference of diastolic blood pressure was 4.26 with a standard deviation of ± 7.35 (*p-value 0.000*), this shows that there is greater variability of the measurements when automated devices are used on hypertensive patients. Van Popele et al reported that increased arterial stiffness causes higher SBP and DBP readings on oscillometric devices although the underlying mechanism of why it occurs is not clear [32]. In our study, both systolic and diastolic readings were overstated majority of the times in comparison to the popular notion that automated devices underestimate readings [33].

An automated device is only recommended if AAMI criteria are fulfilled; that is both systolic and diastolic measurements should not have a mean difference > 5 mmHg or a standard deviation > 8 mmHg. The British Hypertensive Society denotes a grade of A or B if a device is approved [34, 35]. The device we used was the Dinamap Procare 100, which has been validated using both protocols and is on the list of approved devices. Despite this fact, when tested in the emergency setting, it failed to reproduce the same results as was expected for systolic readings. However, it can be stated that this device is capable of measuring systolic and diastolic blood pressure when used on the right arm of non-hypertensive patients. A review by Wan *et al* showed that most of the devices approved by the BHS and AAMI are able to reproduce the results during protocol testing (i.e. artificial settings) with 60-86% of the measurements collected within the reference range of 5 mmHg. However, in devices tested in community/clinic-based studies only 35-46% values were within 5 mmHg of the observed value [36]. Wan *et al* stated that the poor performance in community settings is because the devices do not perform up to par when they are tested outside the validation setting (ideal setting). This is an evident finding in our study, where only 33% of systolic measurements and 44.5% of diastolic measurements were within the range. Skirton *et al* suggested that hypertensive patients, patients with arrhythmias and trauma should be monitored with manual meters as opposed to automated. In our study, in 89 patients with diagnosed HTN, 24.7% of systolic measurements and 39.3% of diastolic measurements were within reference range [19]. The decrease in the validation percentage for hypertensive patients was in accordance with Skirton *et al* [19].

Health care is a rising burden on governments across the world. This is further magnified when developing nations are taken into consideration. There are 28 recommended devices that are considered to be reliable by the British Hypertension Society, which roughly range from 136 GBP to 2000 GBP [28]. Out of these, the prices of 15 devices are listed. The average price is 1258.4 GBP, which equates to PKR 198409.15. The cost is significantly high for countries like Pakistan where 12.7% of the population is below the poverty line [37]. In Pakistan, most of the healthcare is provided by the private sector leading to patients possibly being confronted by catastrophic expenses. More devices are needed which are accurate but also cost effective. Banning mercury sphygmomanometers in third world countries seems a remote possibility until and unless cheaper alternative devices are developed. Another alternative is the use of aneroid BP apparatus, but they need regular calibration, which also at times require mercury sphygmomanometers.

In conclusion, mercury is an environmental hazard and will probably face a worldwide ban as more awareness spreads, thus urgent research for developing new and accurate devices is warranted. Furthermore, separate protocols and criteria have to be established for the use of automated devices in different departments, as the ideal conditions in which these devices are tested won’t be available when these devices are put to test on the field. Our study shows that systolic readings from a previously validated device are not reliable when used in the ED. Secondly, if the blood pressure is measured for the right arm then there is a higher chance of an accurate reading. Lastly, even validated devices show great variability and low precision when they are used outside validation setting. Thus the role of automated blood pressure monitors should be evaluated for their use in the emergency departments.

Awareness needs to be spread amongst physicians that there is a higher level of discrepancy when using these devices on hypertensive patients. Researchers are required to test aneroid blood pressure monitors and there is an urgent need for developing low cost but reliable automated blood pressure devices so that the transition of banning mercury sphygmomanometers is smooth.
